# **Electrophysiological activity in** ***Pinus halepensis*****: a consistent electrical potential relationship between woody and needle tissues**

**DOI:** 10.1080/15592324.2025.2610509

**Published:** 2025-12-30

**Authors:** David Fuente, Rodolfo Zapata, Jose-Vicente Oliver-Villanueva

**Affiliations:** a ITACA - Institute of Information and Communication Technologies, Universitat, Politècnica de València, València, Spain

**Keywords:** Electrophysiological activity, electrical signals, wildfire risk assessment, tree water stress, mediterranean forests, pinus halepensis, leaf fuel moisture content

## Abstract

This study investigates the electrophysiological activity of *Pinus halepensis* to determine whether electrical responses differ among tree organs. Weekly bioelectric voltage measurements were conducted over one year in fifteen trees located in Gátova (Valencia, Spain), comparing electrical potentials between woody (trunk and twigs) and fine tissues (needles). Stainless-steel and platinum electrodes were used to record voltage signals, which were analyzed through linear regression and mixed-effects models. Results showed that voltages in the trunk were consistently higher than in the needles, yet both exhibited synchronized seasonal dynamics driven by shared physiological and environmental factors. The needle-to-trunk voltage ratio remained stable at approximately 60%, except during a summer drought, indicating coherent electrical coupling across organs. A strong linear relationship (R² = 0.98) confirmed that trunk signals serve as reliable surrogates for needle potentials. Organ-level analysis revealed a clear voltage hierarchy (trunk > twig > needle), largely attributable to anatomical and impedance differences. These findings identify the trunk as the optimal electrode placement site, enabling robust, non-destructive, and continuous measurements that can support future applications in wildfire risk assessment and forest monitoring.

## 1. Introduction

Mediterranean forests experience annual drought periods during the summer. Water stress, resulting from scarce rainfall combined with low air humidity and high temperatures, significantly increases vegetation fire risk.^1^ Wildfire plays a key ecological role in Mediterranean ecosystems, although its impact largely depends on fire intensity and recurrence. Indeed, vegetation recovery is slow and thus, recurrent fires can severely hinder natural regeneration.^2-6^ It has been reported that climate change increases the frequency and magnitude of wildfires.^7^ Moreover, climatic and ecophysiological models^8-10^ predict increasingly hot and dry conditions, which heighten the risk of fire outbreaks. However, fire occurrence depends not solely on weather conditions but also on vegetation flammability, which is related to leaf and plant hydration.^11-14^ In this context, fine live fuels—such as leaves, needles, and small twigs that rapidly lose moisture under drought—play a pivotal role in determining ignition probability and fire spread. Therefore, assessing fire risk through the study of fine live fuels—an active area of research^15-19^—is of particular importance.

Increasing attention has been given to analyzing electrophysiological signals generated by plants to understand their physiological processes and internal communication mechanisms.^20-24^ The electrical potential measured in trees reflects the integrated electrochemical state of living tissues, which is tightly coupled to ion transport, water fluxes, and membrane activity. Because these processes are strongly modulated by plant water status, electrophysiological signals provide a sensitive indicator of physiological responses to water stress.^25-27^ Such insights are instrumental in developing more precise predictive fire risk models, thereby enhancing forest management strategies and practices. Studies in plant electrophysiology are normally conducted by inserting metal electrodes into the woody part of the trees,^24-27^ which allows a relatively simple collection of electrophysiological data, especially the electrical potential. Alternatively, other scientists have also measured electrophysiological signals in non-woody parts of the plant.^28-32^ However, while correlations between electrophysiological activity and moisture have been demonstrated,^24^ these relationships have so far been established only for signals measured in woody tissues, not in non-woody components. However, fine live fuels largely determine both the ease of ignition and the rate at which fire spreads through the canopy, shrub and litter layers.^33^^,^^34^ From this perspective, structural and functional differences between tissues^35^ could lead to significant variations in the gathered measurement. For instance, woody and non-woody plant tissues may exhibit markedly different water retention capacities^36^^,^^37^ and electrical properties.^32^^,^^38^^,^^39^

The objective of this research is to improve the understanding of plant electrophysiology by analyzing voltage signals measured in different plant organs. Specifically, we examine whether electrophysiological signals recorded from fine, non-woody tissues differ systematically from those measured in woody tissues, reflecting underlying structural and functional differences. While these electrical measurements alone do not provide direct information on wildfire risk, they capture physiological responses associated with plant water status. As such, this work provides a comparative physiological perspective on electrophysiological measurements in woody and non-woody tissues, which may inform future studies combining electrical signals with additional indicators of plant condition.

## 2. Methodology

a) Why *Pinus* halepensis?

*Pinus halepensis Mill*. is the most widespread and abundant pine species in the western Mediterranean basin, occupying roughly 7 million hectares.^40^ It occurs throughout the Mediterranean region—from Spain and Morocco in the west to southern France, Italy, Greece, Turkey, and Lebanon, in the east, and across North Africa in Algeria, Tunisia, and Libya. Bioclimatic envelope models suggest that the suitable climatic zone for *Pinus halepensis* will continue to expand under future climate scenarios.^41-43^ This species was selected for the present study because of its ecological dominance and representativeness in Mediterranean-type ecosystems.^44^ It is also the main contributor to total biofuel availability in these forests.^19^ Moreover, *P. halepensis* exhibits limited seasonal variation in Leaf Fuel Moisture Content (LFMC),^19^ suggesting that changes in its electrophysiological activity may serve as a more sensitive indicator of phenological changes and drought stress^24^ than the LFMC in this species.

b) Parcel selection

Following previously published works,^24^^,^^45^^,^^46^ the study was conducted in a representative young forest dominated by *Pinus halepensis* (of 93% of total composition), naturally regenerated after a major wildfire. The site, representative of Mediterranean-type forests, is located within the protected area of the Sierra Calderona Natural Park in Gátova, Valencia (Spain), positioned at latitude 39°45′28.8″ N and longitude 0°30′36.36″ W.

The uniform post-fire regeneration following the 1994 wildfire minimized inter-individual variability, an important factor given that tree age can influence the amplitude of electrical signals.^24^ Additionally, the site exhibits consistent edaphic and topographic conditions (soil type, moisture availability, orientation, and slope). A map and an image of the research plot containing the selected fifteen trees can be found in.^46^

c) Tree selection

The stand has a population density of 484 trees/ha, a mean diameter at breast height (DBH) of 12.10 ± 3.95 cm, an average height (h_t_) of 5.16 ± 1.3 m, and an age of 27 years. It was selected for its structural homogeneity, even-aged condition, and absence of recent natural disturbances such as fires, pest outbreaks, or wind damage.

Fifteen trees were selected from the even-aged *P. halepensis* stand following the representative sampling method proposed by Hapla and Saborowski.^47^ This approach ensures that the selected trees are morphologically and physiologically representative of the forest population, thereby enhancing the ecological relevance and generalizability of the results. The same methodology has been successfully applied in previous studies^24^^,^^45^^,^^46^ examining both structural and physiological traits of *P. halepensis* and wood physical properties, ensuring comparability and methodological consistency across studies.

d) Field measurements

The electrophysiological signal of plant tissues was quantified as the voltage difference between the measuring and the reference electrode, expressed in volts (V). Measurements were taken weekly at the hour of zenith in the work area every Sunday for one year, from May 28, 2024, to May 27, 2025. This consistent sampling time minimized diel variability and ensures comparability across dates.^24^^,^^45^^,^^46^ The one-year period allowed the observation of complete seasonal cycles and the assessment of long-term electrophysiological trends. For each of the fifteen sampled trees, two voltages were recorded: one from the trunk and one from the needles, enabling direct comparison between woody and non-woody tissues.

**Figure 1.**
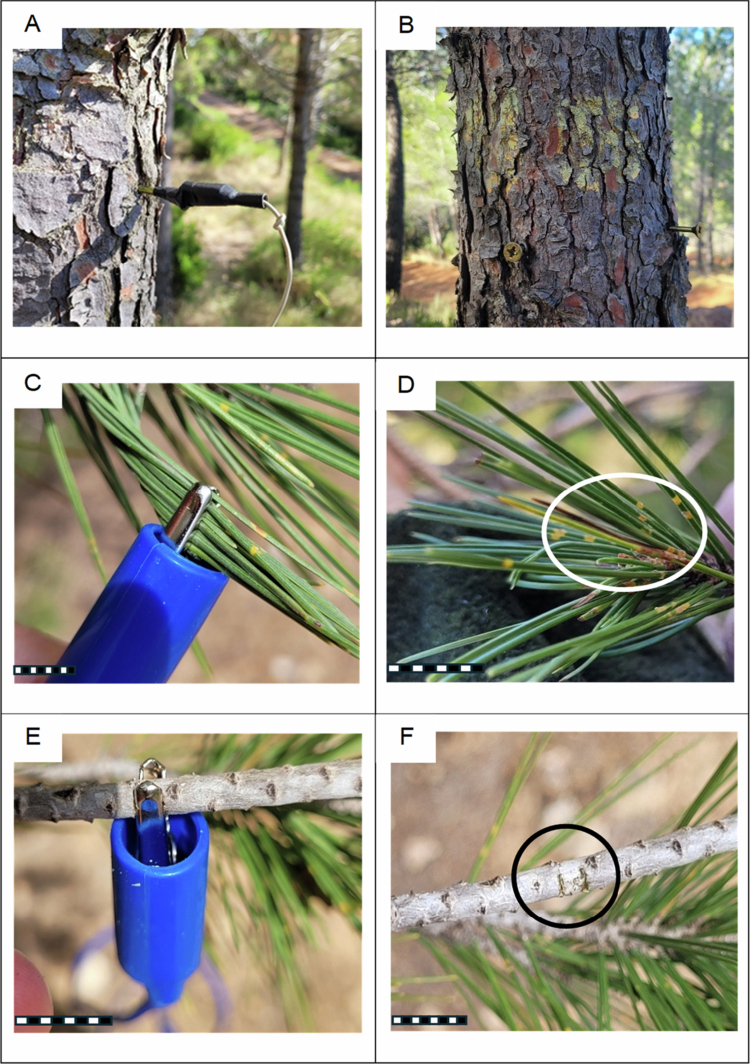
A-B) Stainless steel electrodes were inserted directly into the trunk at 1.5 meters above the ground level, ensuring effective contact with the phloem. C) Placement of the needles inside the pressure elements of a stainless-steel clamp for voltage measurement. D) The mark left by the clamp after taking measurements on the needles appears in yellow. E) A clamp positioned on the twig, near the base where the needles emerge. F) Mark left by the clamp after taking measurements. (scale bars in mm).

Electrophysiological activity in the woody tissue was measured using screw-shaped stainless-steel electrodes (AISI 316, 5 mm thread diameter and 70 mm length) inserted into the tree trunk at a height of 1.5 m above the ground and placed on the north-facing side (Figure 1A-B). Screw-shaped electrodes were chosen for their ease of insertion and removal, ensuring stable electrical contact while minimizing tissue damage. These electrodes have similar characteristics as those employed in our previous studies^24^^,^^45^^,^^46^ and in studies by other authors.^26^^,^^48^^,^^49^ A torque wrench was used to determine electrode contact with the phloem, which was detected by a marked increase in torque upon reaching the xylem interface. This abrupt change in resistance, calibrated in preliminary tests at 1.5 Nm, confirmed a correct positioning without excessive penetration, as the soft phloem requires less torque than the more lignified xylem. This step was essential, as the phloem provides a preferential conductive pathway for bioelectric signals due to its living cells and relatively lower electrical resistance compared with other tissues.^27^ Before each measurement session, the multimeter was calibrated in an open circuit to eliminate background electrical noise. Typical interference levels were consistently below 0.1 mV, while physiological signals ranged from 20 to 1200 mV, ensuring a high signal-to-noise ratio.

For the needle measurements, direct insertion of electrodes was technically not feasible due to the tissue’s thinness. Instead, Several needles were carefully placed, maximizing tissue contact with the electrode, giving priority to filling the space between the jaws of stainless steel clamp with a contact area of approximately 6 mm² and a pressure of 3 N. This method guaranteed stable contact while causing minimal damage to the tissues (Figure 1C). The resulting contact footprint is readily observable by visual inspection (Figure 1D).

A non-polarizable platinum reference electrode was installed in the mineral soil at a depth of 20–25 cm after removing the superficial organic layer. Notably, the hard and rocky soil conditions at the site prevented the installation of the reference electrode at greater depths. The same reference electrode was used for all measurements, and its resistance was checked weekly and consistently remained below 4 Ω, which minimized potential errors in voltage measurements. Electrodes were connected to a high-impedance digital multimeter (UT71D, UNI-T) featuring an input impedance of 2.5 GΩ and a measurement accuracy of ± 0.1% or ± 2 mV, allowing precise voltage acquisition with minimal artefacts. All connections were made using insulated copper cables (0.5 mm diameter and 2 m length) enclosed in flexible plastic sheaths (CE 0123) to prevent external interference and mechanical wear. All reported values correspond to weekly single-point (spot) measurements.

After several months of detailed analysis of electrical variations recorded in both the needles and trunks of the studied trees, we aimed to investigate the observed differences further. We hypothesized that this variation could be attributed to differences in cellular structure and organization between the woody tissues and the needles.^50^ Therefore, to further assess how different tree organs influence the electrical signal, we extended the measurements to include an additional organ—the twig—and conducted spot recordings from the trunk, twig, and needle (Figures 1E–F). All measurements were taken relative to the common soil reference electrode and followed the same calibration protocol as previously described.

Measurements in the twig were carefully performed by placing a thin section between the jaws of a steel clamp, as shown in Figure 1E. The clamp was applied deeply enough to reach the phloem, leaving the mark visible in Figure 1F, which is slightly larger than that shown in Figure 1D (yellow marks).

e) Statistical analyzes:

Linear mixed-effects model of needle-to-trunk voltage ratio over time.

To evaluate whether the electrophysiological relationship between the trunk and needle signals remained stable throughout the year, we analyzed the weekly needle-to-trunk voltage ratio (expressed as a percentage). Since multiple measurements were recorded from the same trees across time, a linear mixed-effects model (LMM) was used to account for repeated measures and non-independence among trees. Date was treated as a fixed effect, and tree identity was included as a random intercept to account for baseline differences among trees. This model has the form:

$${\text{Ratio}}_{\mathrm{ij}}=\beta_{0}+\beta_{1}({\text{Date}}_{j})+b_{i}+\varepsilon_{\mathrm{ij}}$$

where ${\text{Ratio}}_{\mathrm{ij}}$ is the needle/trunk voltage ratio of tree *i* on date *j*, $\beta_{1}$ estimates the fixed effect of time, $b_{i}∼N(0,\sigma_{\text{tree}}^{2})$ is the random tree effect, and $\varepsilon_{\mathrm{ij}}∼N(0,\sigma_{\text{residual}}^{2})$ is the residual error. Mixed-effects models were fitted using the lmer function in the lme4 package in R. Model assumptions were verified by visual inspection of residuals vs fitted plots and normal Q–Q plots. Marginal and conditional R² values were also computed to quantify the variance explained by fixed effects (Date) alone versus the combined fixed and random effects (Date and Tree).

**Figure 2.**
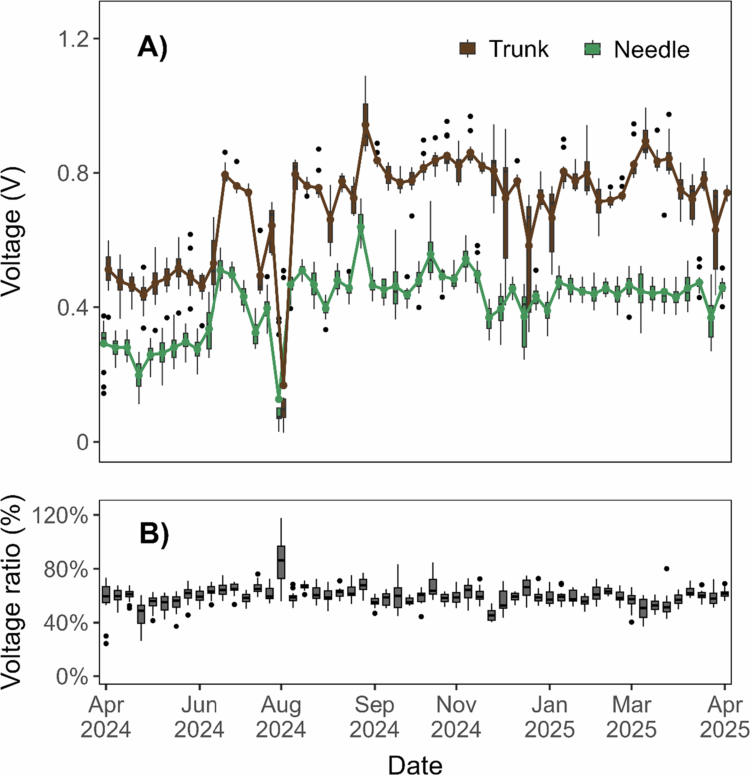
A) This panel illustrates the weekly variation of the electrophysiological signals recorded in both organs throughout the year (52 weeks). It shows the electrophysiological signal (in volts) recorded in the trunk (brown) and needles (green) of the fifteen Pinus halepensis trees. B) Box plots illustrate the percentage ratio between needle and trunk voltages for the same trees. All measurements are reported relative to the common soil reference electrode. Error bars represent ± 1 standard deviation unless indicated otherwise.

One measurement date in August 2024, that is, during the week with the most intense hydric stress, exhibited a markedly elevated mean voltage ratio (85.8%) and was identified as an outlier using exploratory descriptive statistics —specifically, a visual spike in the time series and an anomalously high mean relative to any other data (Figure 2B). To determine whether this event influenced model behavior, the analysis was conducted both with and without this date. This approach is consistent with ecological statistical practice where transient extreme events are tested separately from baseline physiological trends.

The primary hypothesis tested was whether the ratio remained constant across dates, i.e., whether Date exerted a statistically significant fixed effect on the needle/trunk voltage proportion.

Linear regression model between the voltage at the trunk and at the needles.

A linear regression model without intercept (Needle ~ 0 + Trunk) was applied to assess the proportional relationship between trunk and needle electrophysiological signals in R. The intercept was constrained to zero based on the biophysical expectation that both voltages approach zero under electrically neutral conditions. Model residuals were evaluated for normality via Q–Q plots and for homoscedasticity via residual vs fitted plots, and no meaningful violations were detected. The coefficient of determination (R²) and regression slope were used to quantify the strength of coupling between organs. Root mean square error (RMSE) and 95% confidence intervals of the slope were calculated to assess prediction accuracy and parameter uncertainty.

Linear mixed-effects model of the organ-specific voltage differences and the tree effect.

To assess whether electrophysiological voltage differed among tree organs and whether these differences were influenced by the tree specimen, we used a linear mixed-effects modeling (LMM) framework. Voltage measurements were collected simultaneously from the trunk, twig, and needle of each tree. Because repeated observations were obtained from the same individuals, a mixed-effects model was used to account for non-independence among trees. Organ type (Trunk, Twig, Needle) was treated as a fixed effect, and tree identity was included as a random intercept to account for baseline differences among individuals. The model has the form:

$${\text{Voltage}}_{\mathrm{ij}}=\beta_{0}+\beta_{1}({\text{Part}}_{j})+b_{i}+\varepsilon_{\mathrm{ij}}$$

where ${\text{Voltage}}_{\mathrm{ij}}$ is the measured voltage for tree i in organ j, $\beta_{1}$ estimates the fixed effect of organ, $b_{i}∼N(0,\sigma_{\text{tree}}^{2})$ is the random effect of tree identity, and $\varepsilon_{\mathrm{ij}}∼N(0,\sigma_{\text{residual}}^{2})$ is the residual error. The mixed-effects model was fitted using the lmer function in the lme4 package in R. Model assumptions were verified by inspection of residual vs. fitted plots and Q–Q plots. Post-hoc pairwise comparisons among organs were conducted using Tukey-adjusted estimated marginal means via the emmeans package to identify statistically distinct organ-level voltage patterns.

## 3. Results and discussion

The electrophysiological monitoring conducted over one year revealed clear temporal patterns in the electrical activity of *Pinus halepensis.* Figure 2A illustrates the weekly fluctuations of the electrical voltage measured in a population of fifteen Aleppo pines. The dataset includes two components: the electrophysiological signal recorded in the trunk (brown line) and in the needles (green line) of the same trees.

In addition to the overall similarity between organs, Figure 2A shows a pronounced decline in electrical activity during mid-summer (July–August), followed by abrupt recoveries coinciding with rainfall events. Such transient drops and rapid increases are consistent with drought-induced reductions in sap flow and ion transport, followed by hydraulic reactivation after precipitation. Because electrical potentials in trees are tightly coupled to ion fluxes, membrane activity, and water transport in conductive tissues, these temporal patterns reflect physiological adjustments to changing water availability rather than structural damage.^51^

Remarkably, the electrophysiological signal measured in the trunk consistently exceeded that recorded in the needles throughout the entire period. Despite these differences, both datasets displayed similar annual trends, with peaks and valleys coinciding in time. This observation indicates that both potentials are influenced by the same environmental and physiological factors, reflecting a shared electrical activity within the tree.^46^^,^^48^ Figure 2B shows that the ratio between the trunk and the needles potentials remained approximately constant at around 60%, except for a few cases—most notably at the beginning of August. The dispersion of the data, as indicated by the length of the boxes, was generally low in most of the measurements, with few exceptions.

To assess whether the voltage ratio remained constant over time, a mixed-effects model was applied across all sampling data. When all dates were included, the mixed-effects model indicated a statistically significant effect of Date on the ratio (F₁,₇₉₄ = 4.32, *p* = 0.038), driven by a single week in August when the mean ratio rose sharply to 85.8%. This event likely reflects an acute physiological stress episode, such as rapid rehydration or loss of hydraulic conductivity.

To evaluate the baseline physiological pattern, this outlier date was removed and the mixed-effects analysis repeated. After exclusion, the effect of Date was no longer statistically significant (F₁,₇₇₉ = 0.95, *p* = 0.330), indicating no temporal variation in the needle-to-trunk voltage ratio under normal physiological conditions. Residual diagnostics confirmed model validity: the residuals were symmetrically distributed around zero, and residual-fitted plots showed no heteroscedasticity. The Shapiro–Wilk test (W = 0.971, *p* < 0.001) indicated deviations from perfect normality, as expected in large ecological datasets, though the QQ plot showed these deviations were confined to the tails and within acceptable limits for linear-mixed modeling. In summary, the needle-to-trunk voltage ratio remained statistically stable across the year under non-extreme conditions, reflecting a consistent electrophysiological coupling between tree organs. The transient increase in August represents a biologically meaningful stress response rather than random variability. These results suggest that the ratio between the trunk and needle potentials in *Pinus halepensis* constitutes a reliable and stable physiological indicator under the studied conditions.

**Figure 3.**
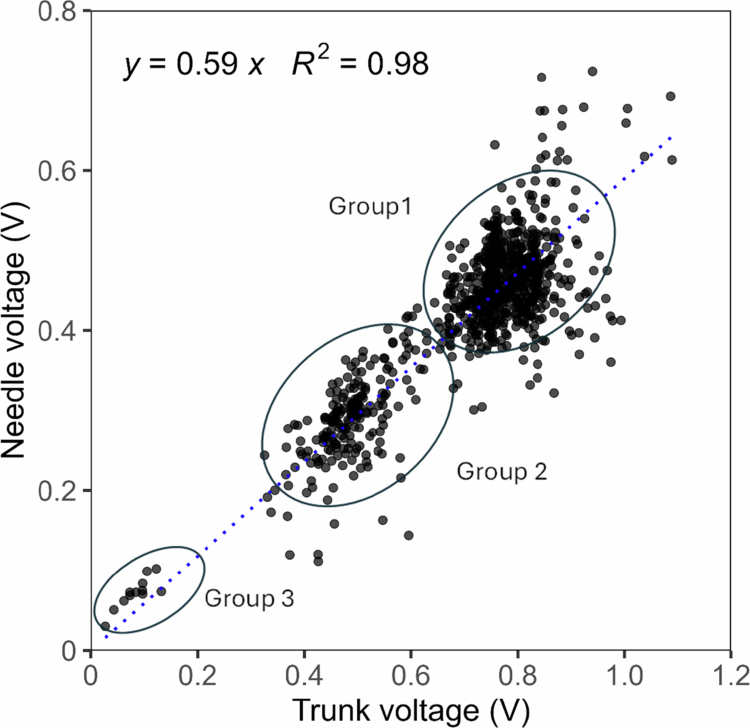
Scatter plot showing the relationship between the electrophysiological signals recorded in the trunks and needles of the Aleppo pines. Each data point represents a simultaneous measurement from both organs of a single tree over one year (52 weeks and 15 trees). The blue dotted line represents the linear regression. The corresponding equation and associated (R^*2*^) value are shown inside the figure.

Figure 3 shows the relationship between the electrophysiological signals recorded in the trunk and in the needles of the trees, obtained from simultaneous weekly measurements over the course of one year. Each data point represents a paired voltage measurement from both organs of a single tree. Needle displayed a strong linear correlation with trunk voltage, following the model Needle = 0.59 × Trunk (R² = 0.98, *p* < 0.001). The regression slope differed significantly from unity (95% CI: 0.57–0.61), indicating that needle voltage consistently accounted for approximately 59% of trunk voltage across individuals and seasons.

Despite the inherent biological variability in the data, residual analysis confirmed no violation of linearity or homoscedasticity. The model’s root mean square error (RMSE) was 0.053 V, representing about 10% of the mean voltage and indicating that prediction errors were small relative to overall variations. The combination of the high R², low RMSE, and narrow confidence interval suggests that trunk voltage acts as a robust integrator of tree electrical activity and can be used as a reliable predictor of needle voltages. This strong linear relationship reveals a coherent electrical signaling network throughout the tree, likely driven by shared physiological processes such as sap flow and ionic fluxes.^26^^,^^27^^,^^49^

Three clusters can be observed in Figure 3, each corresponding to three different periods: favorable growth conditions in autumn and spring (group 1), progressive water stress accompanied by rising temperatures (group 2), and peak unfavorable summer conditions (group 3), which occurred on August 11, 2024. On that day, the maximum temperature reached 32.1 °C, relative humidity dropped to 38%, and wind speed rose to 33.8 km h⁻¹ —conditions indicative of high fire risk and approaching the thresholds of the 30–30–30 wildfire rule (temperatures >30 °C, humidity <30%, and wind speeds >30 km h⁻¹). These clusters likely reflect physiological adjustments to seasonal changes in water availability and stomatal conductance, aligning with previous findings in *Pinus halepensis* under Mediterranean drought cycles.^52^^,^^53^

Overall, the results indicate that the trunk-based electrophysiological measurement can serve as a reliable proxy for estimating the needle voltage. Trunk recordings are technically simpler, minimally invasive, and more stable, making them suitable for long-term monitoring of tree activity. Notably, as concurrent LFMC and high-frequency meteorological data were not collected, the present study cannot establish a direct relationship between electrophysiological signals, fuel moisture, and fire-risk indicators. Consequently, our conclusions are just limited to organ-level voltage comparisons.

**Figure 4.**
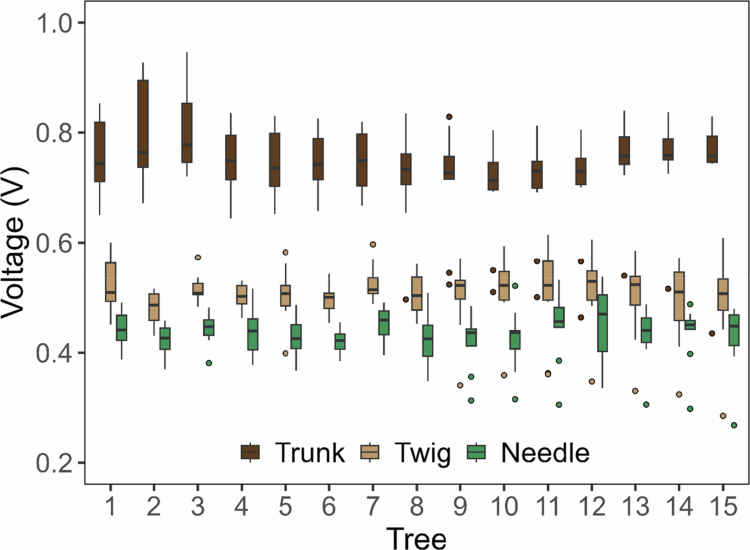
Weekly electrophysiological measurements from three parts of fifteen trees in the study population during the first four months of 2025 are shown as box plots. Trunk voltages are represented in light brown, voltages at the twigs in beige, and needle voltages in green.

Figure 4 presents the electrophysiological voltage recorded from the studied pines during the initial four months of 2025. Measurements were taken at three parts of each tree: the trunk (brown), the twig (beige), and the needles (green). Measurements in the trunk were obtained by inserting an electrode directly into the wood, thereby enabling direct contact with the phloem—a tissue rich in conductive sap. In contrast, the electrode insertion at the needle base and within the needles was not technically feasible, so stainless-steel alligator clips were used instead (Figures 1C and 1E). This setup provided a reduced electrical contact, increasing contact resistance. The data reveal a clear and consistent potential gradient across all trees. The highest electrical potentials were consistently recorded in the trunk, ranging from 0.7 to 0.9 V. Intermediate values, between 0.5 and 0.6 V, were measured at the needle base or twigs, while the lowest potentials, from 0.4 to 0.5 V, were observed in the needles.

The differences among these voltages likely arise from both anatomical constraints and variations in electrode-tissue impedance. Needles have thinner cuticles, lower sap volume, and higher resistance, which limit signal amplitude.^54^^,^^55^ In contrast, inserting the electrode into the trunk allows direct contact with sap-rich, highly conductive tissues and provides a larger electrode-tissue interface, improving signal transmission. Conversely, using alligator clips for the needles and their base results in shallower contact, higher resistance, and lower measured electrical potentials. Furthermore, the limited sap volume and smaller tissue thickness of the needles further contribute to their reduced electrical signal.

To statistically evaluate the voltage differences among organs, a linear mixed-effects model was applied to the voltage data. Electrophysiological voltage differed significantly among the three measured organs—trunk, twig, and needle. The mixed-effects model revealed a strong fixed effect of organ (F₂,₄₃₃ = 898.67, *p* < 0.001), with organ type explaining 80.0% of the total variance (marginal R² = 0.8001). Estimated marginal means (±95% CI) confirmed a clear and consistent voltage hierarchy: trunk voltage was the highest (0.742 V; 95% CI: 0.731–0.753), followed by twig voltage (0.506 V; 95% CI: 0.495–0.516), and needle voltage was the lowest (0.434 V; 95% CI: 0.424–0.445). All pairwise contrasts were significant (Tukey-adjusted *p* < 0.001), indicating that each organ maintained a statistically distinct electrophysiological potential.

Tree individuals did not substantially influence the absolute voltage measured in each organ. The random-intercept variance was extremely small (SD = 0.00047 V), and the conditional R² (0.80012) was nearly identical to the marginal R² (0.80011), demonstrating that inter-individual variation in baseline voltage levels was negligible relative to organ-level effects. Thus, trunk, twig, and needle voltages were highly conserved across trees, indicating a species-level pattern.

## 4. Conclusions

This study provides evidence of a strong and consistent relationship between the electrophysiological signal recorded in the trunk and the needles of *Pinus halepensis*. The main conclusion is that both organs exhibit comparable electrophysiological variations, characterized by similar temporal patterns in response to physiological and environmental drivers. This finding confirms that trunk-based measurements can serve as reliable proxies for monitoring whole-tree electrophysiological activity and, by extension, for assessing physiological water stress and potential wildfire risk.

The consistent voltage ratio observed between trunk and needle measurements primarily reflects differences in electrode–tissue contact quality and in the anatomical and hydraulic characteristics of each organ. Moreover, the practical limitations of needle-based recordings—stemming from their small size, structural fragility, and reduced electrode stability—underscore the advantages of trunk-based measurements for long-term, in situ monitoring. The trunk represents an optimal site for electrode installation, providing better electrical contact, lower impedance, and greater repeatability across measurements and environmental conditions.

Future research should investigate whether the relationship between trunk and needle voltages remains consistent across other species and contrasting environmental scenarios. Implementing systematic and automated acquisition of large, high-resolution datasets using advanced data capture systems (DAQs) with real-time and remote capabilities will be essential. In parallel, the integration of electrophysiological data with LFMC and microclimatic measurements aims to demonstrate that electrical signals can quantify key inflammability-related indices and be effectively incorporated into fuel and wildfire risk models.
